# Digital strolls using UniCDent toolkit-part 2: reimagining the walking method for online participation

**DOI:** 10.3389/froh.2025.1545214

**Published:** 2025-06-18

**Authors:** Prashanti Eachempati, John Martin, Sally Hanks, Mona Nasser

**Affiliations:** ^1^ Peninsula Dental School, University of Plymouth, Plymouth, United Kingdom; ^2^ Faculty of Dentistry, Manipal University College Malaysia, Melaka, Malaysia; ^3^ School of Geography, Earth and Environmental Sciences, University of Plymouth, Plymouth, United Kingdom; ^4^ Faculty of Health, University of Plymouth, Plymouth, United Kingdom; ^5^ Peninsula Dental School, University of Plymouth, Plymouth, United Kingdom

**Keywords:** participatory research, UniCDent toolkit, online engagement, digital walking method, remote participation

## Abstract

Distance and remoteness no longer pose barriers to conducting meaningful research, as the adaptation of participatory methods can address these challenges effectively. This article examines the transformation of the UniCDent Toolkit, originally designed to capture patients' perceptions of uncertainty in dental environments, for online participatory interactions with dentists. The toolkit, which uses the walking method, incorporates components such as imagery, gallery walks, quadrant mapping, and trade-offs to explore uncertainty in dental practice. Initially, dentists expressed discomfort in sharing their uncertainty in a group setting, prompting a shift to an online format that maintained participant engagement and created a safe space for open dialogue. Each component was carefully tailored for the virtual setting: dentists documented their uncertainty using auto-photography, shared insights through a structured slide walk, collaboratively mapped their uncertainty with a virtual grid, and participated in trade-off discussions using Mentimeter polling. This process highlights the importance of adapting participatory methods to meet the needs of remote participation, while preserving the participatory ethos. We provide an example of such an adaptation, demonstrating how the UniCDent Toolkit which was initially designed for dental environments can be applied to various healthcare settings and research questions.

## Introduction

Participatory research methods have emerged as powerful tools in community-based health research, fostering active engagement and collaboration between researchers and participants (1). One such approach, the walking method, draws from the idea that immersive experiences in specific environments can lead to richer insights into participants' perceptions and lived experiences (2). By encouraging individuals to interact directly with their surroundings, the walking method allows for sensory engagement, promoting reflection and providing valuable data often overlooked by traditional methodologies (3). As participatory research moves into remote and virtual spaces, these methods must evolve to accommodate distance without losing their immersive quality, demonstrating that meaningful engagement can still occur across geographical and contextual boundaries.

This participatory ethos inspired the development of the UniCDent Toolkit—an acronym for “Uncertainty in Clinical Dentistry Toolkit.” Originally designed to explore how patients perceive uncertainty while navigating dental environments, the toolkit employs auto-photography and photo elicitation in conjunction with the walking method to elicit insights about patients' experiences (4). This innovative approach is detailed in another article, which focuses on the data collected from patients regarding their uncertainty in clinical settings (5).

However, challenges arose when we sought to adapt the UniCDent Toolkit for dental practitioners. In this case study, we showcase how the UniCDent Toolkit was adapted for remote use, illustrating the flexibility of participatory methods in overcoming the challenges of distance and remoteness.

Although originally designed for exploring uncertainty in dental environments, the toolkit's adaptable components can be applied in various healthcare settings and used to answer different research questions. This example demonstrates how participatory research can evolve to suit diverse contexts while maintaining its core principles of engagement and collaboration.

### The UniCDent toolkit components

The UniCDent Toolkit comprises four key components (5) that facilitate a structured approach to understanding and addressing the complexities of uncertainty in dental practice:

### Imagery

Participants use auto-photography to capture their dental environment, focusing on elements that evoke feelings of uncertainty. This visual documentation encourages self-reflection and helps identify specific sources of concern, as patients navigate the dental environment and click photographs.

### Gallery walk

This is an interactive activity where participants share photographs and explain why they took them; this component promotes dialogue among participants about the uncertainty represented in their images. Patients printed their photographs, displayed them on a string, and explained their significance to foster the exchange of insights and experiences.

### Quadrant mapping

This exercise allows participants to categorise their uncertainty based on perceived impact on decision-making. Patients mapped their photographs on a grid with four quadrants, representing high and low uncertainty alongside high and low impact on decision-making.

### Trade-offs

In this phase, participants discussed and prioritised strategies for managing uncertainty, creating a collective understanding of strengths and approaches to leverage in practice. Flashcards were provided for participants to choose their top three trade-offs.

## UniCDent toolkit for dentists' participatory workshops

**Figure 1:**
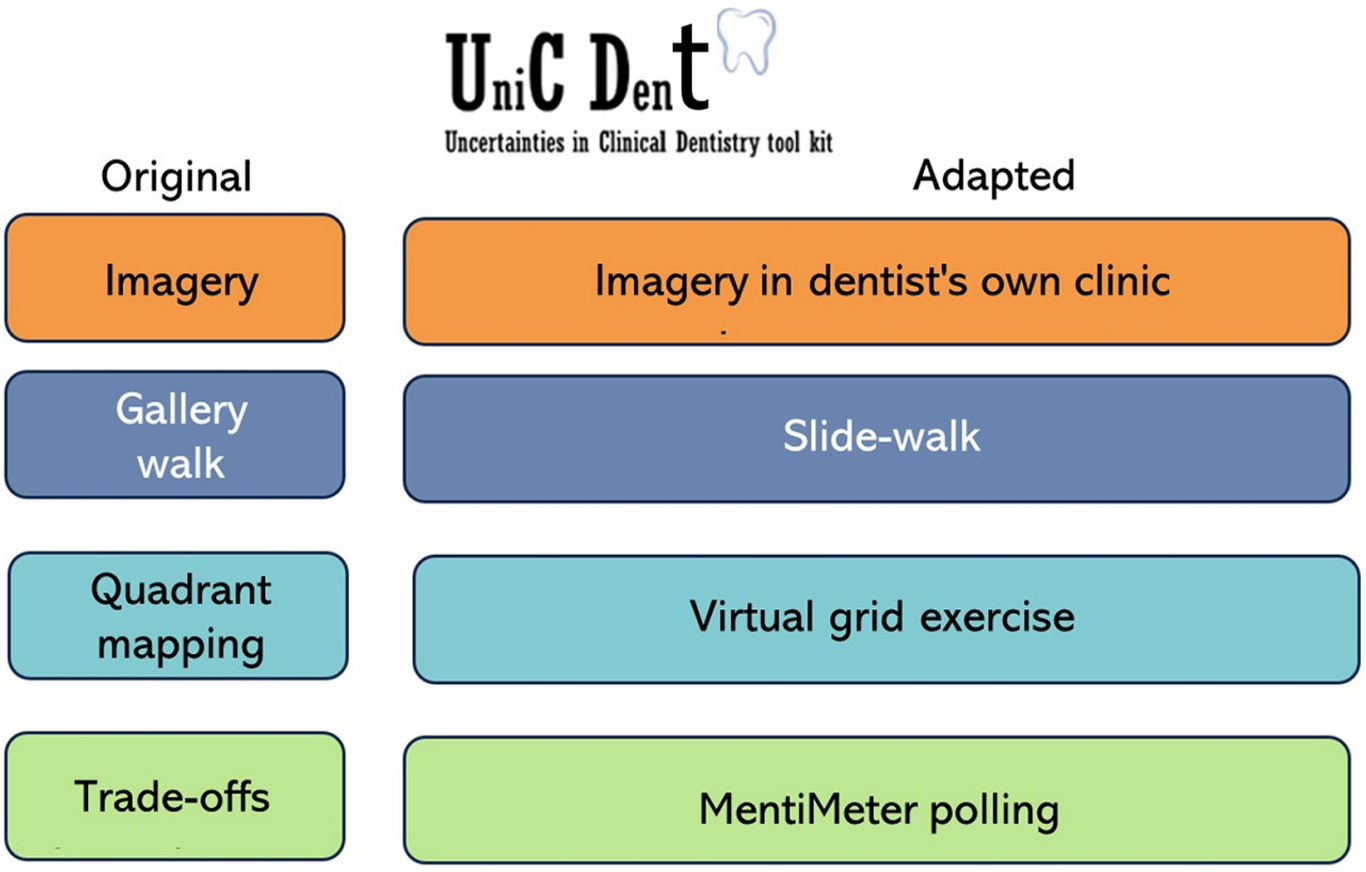
Adaptation of UniCDent toolkit.

Our initial plan was to conduct in-person participatory workshops with 12 Malaysian dentists, each having at least one year of experience in either government or private practice, where they could openly share the uncertainty they face and the trade-offs they make in clinical practice. The data from these workshops was planned to be analysed using reflexive thematic analysis to understand what they perceive as uncertainty and how they respond to it. However, many dentists expressed discomfort in revealing their uncertainty in front of their peers, fearing that such exposure could give “competitors” an advantage. Government dentists, in particular, highlighted concerns about sharing the perceived uncertainty in their clinical environments. As a result, we had to pivot our approach and adapt the UniCDent Toolkit for an online format, ensuring that we maintained participant engagement while providing a safe space for sharing (Figure 1).

## Adapting the UniCDent toolkit for online engagement

### Imagery

The imagery component of the UniCDent Toolkit utilises auto-photography, a participatory research method where participants capture images from their environments that hold personal significance. For our interaction with dentists, we recognised that asking them to photograph uncertainty within a researcher-defined setting, such as my dental clinic, would be irrelevant and unproductive. Instead, we focused on enabling dentists to document uncertainty specific to their own practice settings, providing a true reflection of their day-to-day experiences.

To facilitate this process, we allowed dentist participants 15 days to take photographs whenever they encountered situations that caused feelings of uncertainty. This autonomy encouraged them to engage authentically with their environments, capturing instances that resonated with their individual experiences.

We also addressed potential consent issues, particularly concerning the privacy of patients. To mitigate these concerns, we instructed participants to avoid photographing patients directly. Instead, we encouraged them to either document their reflections in writing or to capture images of objects or settings that exemplified their uncertainty—such as dental tools, clinical setups, or workflows—without compromising patient confidentiality.

### Gallery walk: slide walk

The gallery walk was adapted into a “Slide Walk,” allowing for a structured online interaction that preserved the participatory essence of the original method. Participants were requested to submit their photographs in advance, along with an indication of any specific images they wished to discuss first. This preparation enabled the facilitator to navigate the workshop in a sequential manner, exploring each image based on the dentists' preferences and insights.

**Figure 2:**
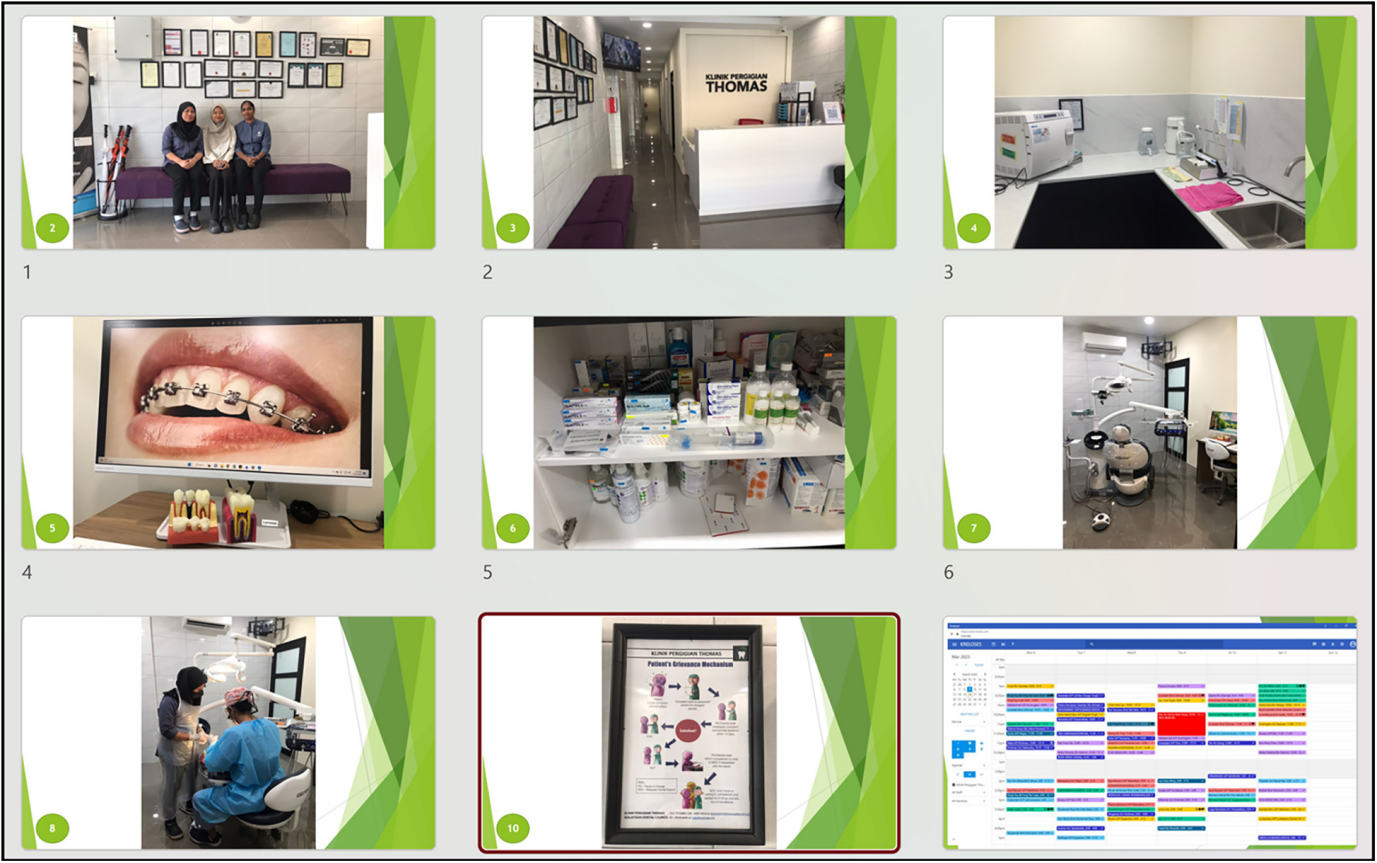
Slide walk- dentist participants explain their photographs, discussing the uncertainty in their environments.

Moving from one slide to another, participants shared their thoughts and experiences related to the images, fostering a rich dialogue about the uncertainty they face in their dental practices (Figure 2). We employed photo elicitation within this framework, a technique that utilises visual mediums to stimulate verbal discussions. This method unveils different layers of meaning by evoking deep emotions, memories, and ideas from participants (4). Research indicates that visual images can engage deeper parts of human consciousness than words alone (6). Although the traditional gallery walk provides a dynamic group setting, this adapted approach ensured that each dentist could voice their perspectives in a focused manner, in a private space enhancing individual engagement.

### Quadrant mapping: virtual grid exercise

**Figure 3:**
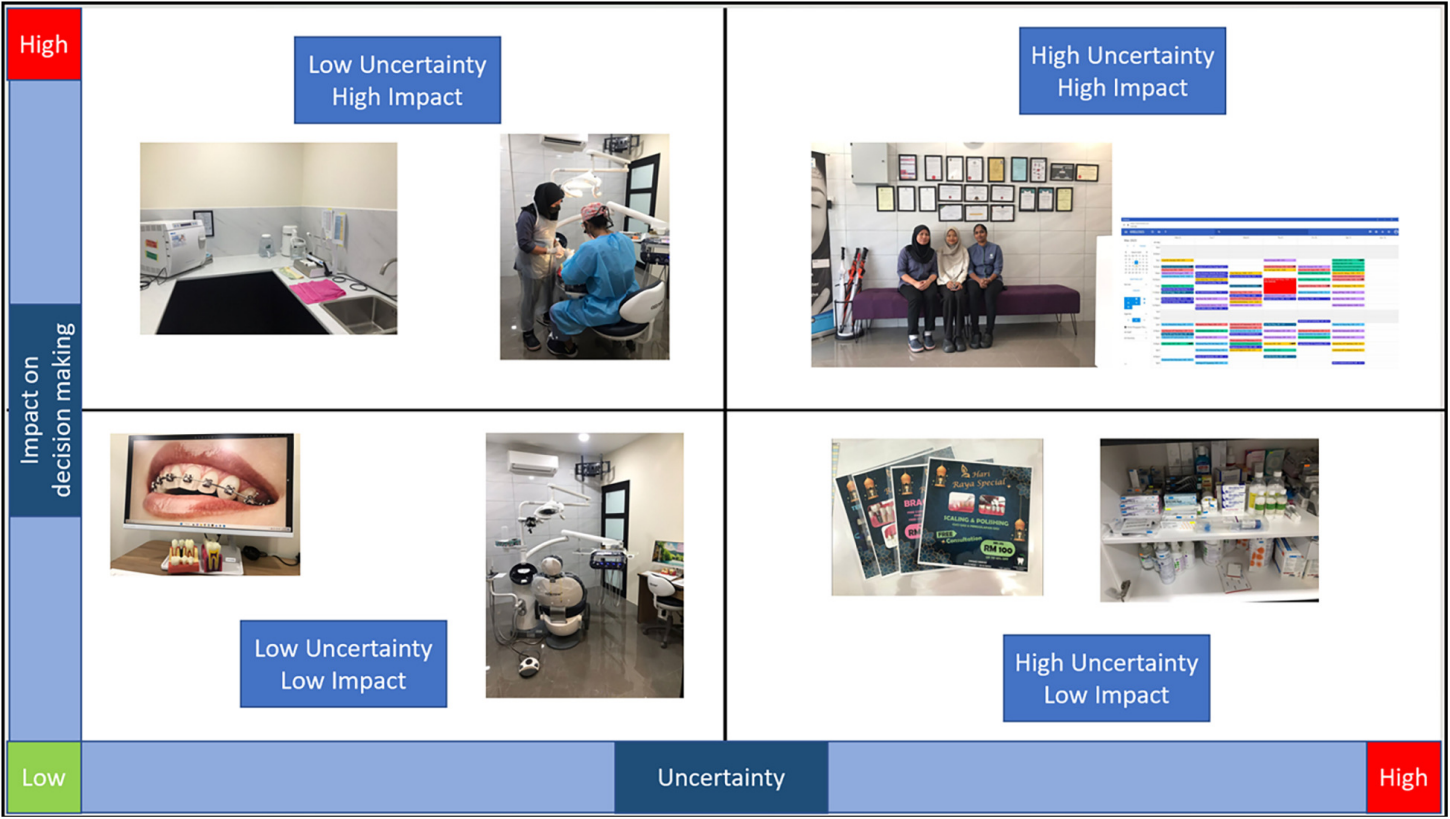
Online grid exercise on OneDrive document.

The quadrant mapping exercise was adapted for a digital format using OneDrive. We created a PowerPoint presentation featuring a grid and shared a link that allowed participants to upload their photographs according to their perceived level of uncertainty and its impact on decision-making. This collaborative approach between participant and researcher facilitated real-time interaction, as participants could place their photos on the grid while the researcher visually monitored the updates (Figure 3).

To enhance engagement, we utilised screen sharing during our discussions, which enabled us to analyse and reflect on the images together as they were added to the grid. The use of a familiar platform like OneDrive made the exercise accessible and user-friendly for all participants, ensuring that they could engage comfortably with the task. This dynamic interaction not only preserved individual autonomy but also allowed the participating dentist to share insights and articulate their experiences with the researcher. However, this approach may limit the opportunity for participants to consider differing perspectives from colleagues, as the impact of others’ feelings or disagreements on individual decisions is minimised. While this can help maintain personal autonomy and clarity, it also means that participants do not engage in collaborative discourse, which could influence how they weigh their decisions. Acknowledging this difference is important, as the absence of peer influence may be seen as both a strength and a limitation depending on the context.

### Trade-offs: Mentimeter polling

**Figure 4:**
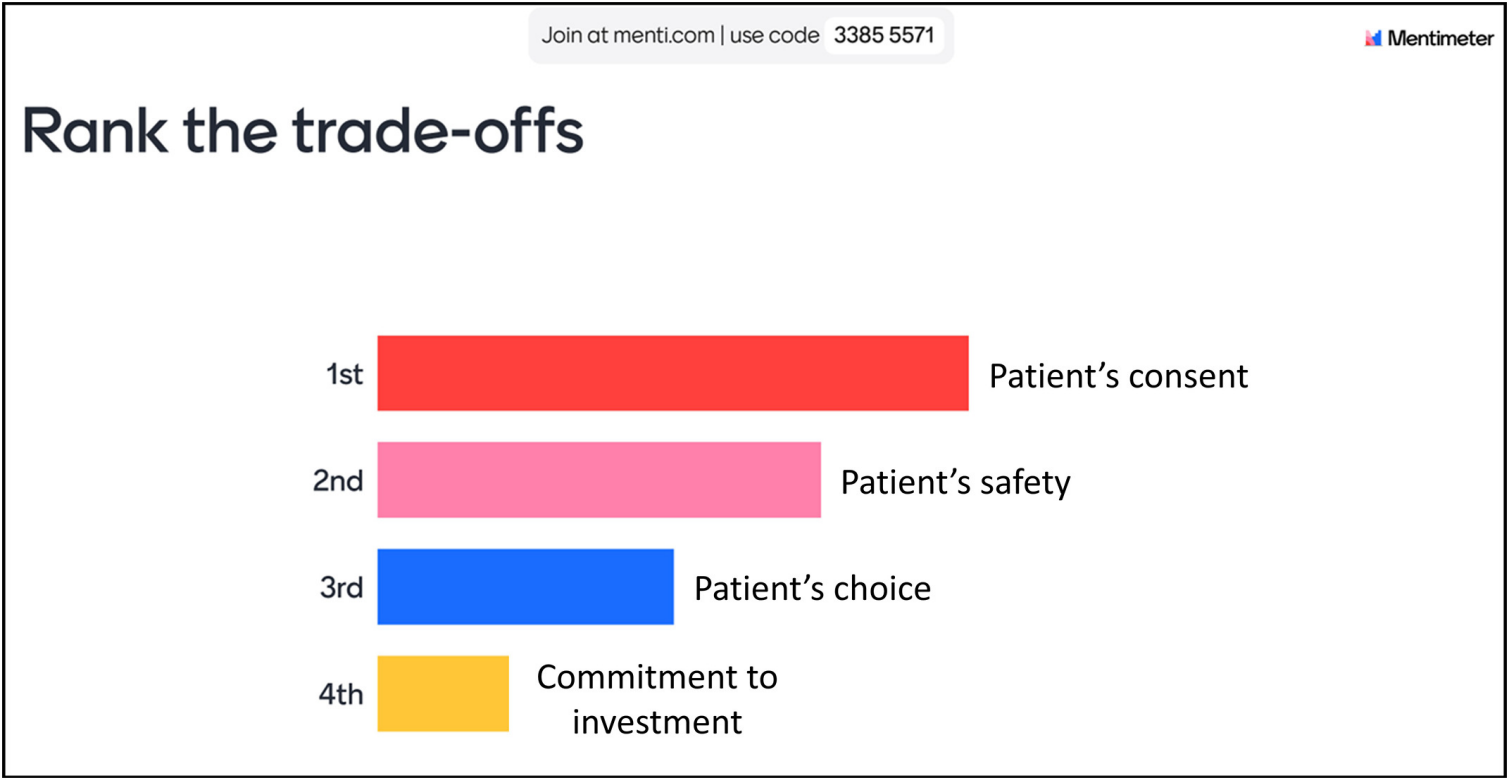
Participants ranking their trade-offs in Mentimeter poll.

**Figure 5:**
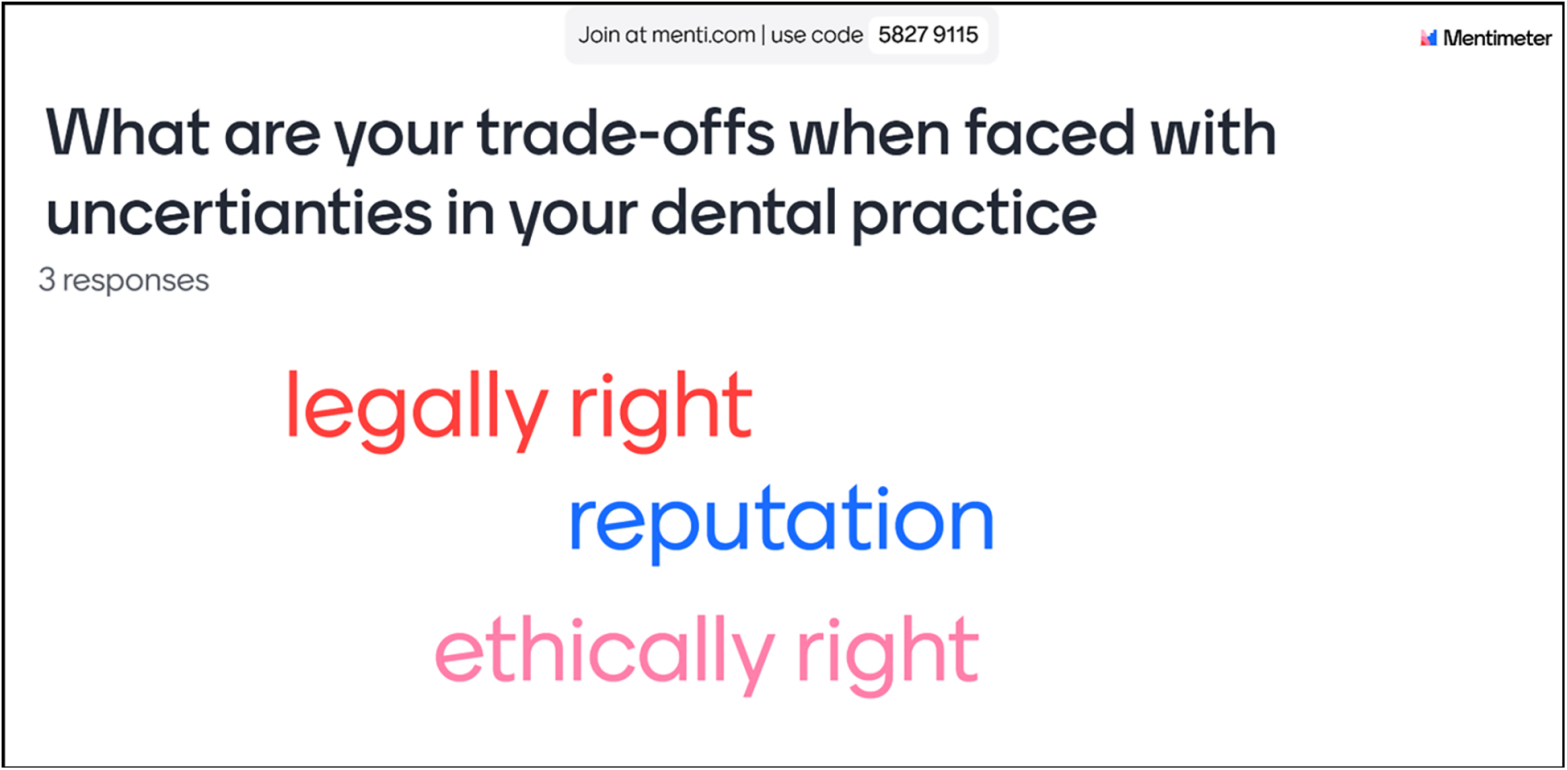
Participants giving own suggestions for trade-offs in Mentimeter wordle.

At the beginning of this project, a focus group discussion was arranged with all dentists before conducting the individual online exercises. During these focus groups, participants were encouraged to voice the common trade-offs they typically face when dealing with uncertainty in clinical practice. For the individual sessions, we employed Mentimeter, an interactive polling tool, allowing the dentists to engage in ranking these trade-offs in real-time (Figure 4). Additionally, participants were encouraged to contribute their own suggestions in a wordle, enabling them to highlight specific trade-offs that resonated with their experiences (Figure 5).

The interactive nature of Mentimeter empowered dentists to express their preferences openly, fostering a sense of ownership over their contributions to the discussion. Using this method allowed participants to see their preferences emerge on the screen, prompting dynamic conversations about why certain trade-offs were prioritised over others.

### Reflecting on this adaptation

Participatory methods, rooted in social constructivism, emphasise that knowledge is co-created through collaboration and engagement among participants (7). This principle highlights the importance of involving participants as active partners in the research process. In adapting these methods for remote participation, we encountered the challenge of maintaining meaningful collaboration in a virtual setting.

Our commitment to the core values of participatory research which are active engagement, inclusivity, and co-creation, remained strong. Recognising that moving to a virtual format could risk reducing the sense of community and collaborative learning, we adapted our approach to encourage active remote participation. We utilised interactive tools such as Mentimeter and OneDrive for real-time collaboration, ensuring participants could engage meaningfully despite the physical distance. These tools fostered dialogue, reflection, and dynamic involvement, allowing participants to express their insights and contribute actively.

By acknowledging the challenges of remote participation and thoughtfully integrating technology to maintain engagement, we ensured that participants remained at the heart of the research process. This experience highlighted the potential of remote participatory methods to overcome geographic barriers and demonstrated how such adaptations can foster meaningful collaboration across various settings and disciplines. The success of these adaptations underscores the broader application of participatory methods in a range of contexts, beyond traditional research environments.

## Conclusion

In conclusion, the adaptation of the UniCDent Toolkit for remote participation illustrates the flexibility and innovation that participatory research methods offer, especially in overcoming challenges of distance and remoteness. By utilising tools like auto-photography, virtual gallery walks, real-time collaboration platforms, and interactive polling, we were able to foster meaningful engagement and collaboration in an online environment. This adaptation demonstrates the broader applicability of participatory methods across diverse settings, ensuring that participants remain active contributors to the research process, regardless of physical location.

## Data Availability

The original contributions presented in the study are included in the article, further inquiries can be directed to the corresponding author.
